# Understanding the anatase–rutile phase junction in charge separation and transfer in a TiO_2_ electrode for photoelectrochemical water splitting†
†Electronic supplementary information (ESI) available. See DOI: 10.1039/c6sc01611a


**DOI:** 10.1039/c6sc01611a

**Published:** 2016-06-09

**Authors:** Ailong Li, Zhiliang Wang, Heng Yin, Shengyang Wang, Pengli Yan, Baokun Huang, Xiuli Wang, Rengui Li, Xu Zong, Hongxian Han, Can Li

**Affiliations:** a State Key Laboratory of Catalysis , Dalian Institute of Chemical Physics , Chinese Academy of Sciences , Dalian National Laboratory for Clean Energy , 457 Zhongshan Road , Dalian , 116023 , China . Email: hxhan@dicp.ac.cn ; Email: canli@dicp.ac.cn; b Graduate University of Chinese Academy of Sciences , Beijing 100049 , China; c Collaborative Innovation Center of Chemistry for Energy Materials (iChEM) , China

## Abstract

New insight into junction-based designs for efficient charge separation is vitally important for current solar energy conversion research. Herein, an anatase–rutile phase junction is elaborately introduced into TiO_2_ films by rapid thermal annealing treatment and the roles of phase junction on charge separation and transfer are studied in detail. A combined study of transient absorption spectroscopy, electrochemical and photoelectrochemical (PEC) measurements reveals that appropriate phase alignment is essential for unidirectional charge transfer, and a junction interface with minimized trap states is crucial to liberate the charge separation potential of the phase junction. By tailored control of phase alignment and interface structure, an optimized TiO_2_ film with an appropriately introduced phase junction shows superior performance in charge separation and transfer, hence achieving *ca.* 3 and 9 times photocurrent density enhancement compared to pristine anatase and rutile phase TiO_2_ electrodes, respectively. This work demonstrates the great potential of phase junctions for efficient charge separation and transfer in solar energy conversion applications.

## Abstract 2

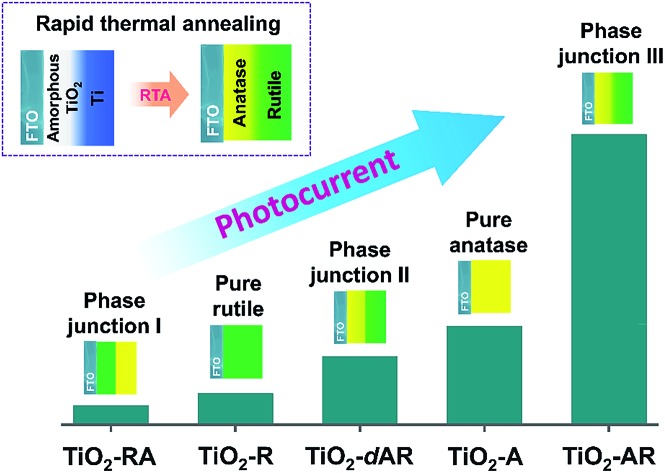
The key to phase junctions for efficient charge separation is to consider both the phase alignment and interface structure.

## Introduction

Separation of photogenerated charges carriers is one of the most crucial factors determining the efficiency of photocatalytic (PC) or photoelectrochemical (PEC) water splitting.^1^–^7^ Various kinds of strategies have been pursued for achieving efficient charge separation and transfer. Fabrication of heterojunctions has been confirmed to be an effective strategy for achieving efficient charge separation and transfer.^3^–^5^ We have also demonstrated that the phase junctions in polymorph semiconductors, such as the anatase–rutile phase junction in TiO_2_ (ref. 6) and the α–β phase junction in Ga_2_O_3_,^7^ can dramatically increase the photocatalytic activities of the corresponding particulate semiconductors due to efficient separation and transfer of photogenerated charges between the different phases.

Since the first demonstration of photoelectrochemical water splitting on a TiO_2_ photoanode,^8^ intensive research has been carried out to understand the effect of the nature of TiO_2_ semiconductor photocatalysts on charge separation and transfer. The unique efficacy of a phase junction in charge separation and transfer has been well demonstrated in particulate TiO_2_,^9^–^11^ which is accountable for the superior photocatalytic performance of commercial Degussa P25 TiO_2_. However, its role is still obscure in photoelectrodes,^8^,^12^,^13^ photovoltaic cells^14^,^15^ and other important film-based energy conversion devices,^16^–^18^ where TiO_2_ films are widely used. One may expect a similar positive performance gain when a phase junction is introduced into these thin-film based systems. However, it has been reported that when designed with no consideration to the alignment of different phases, the electrode deposited with particulate TiO_2_ containing an anatase–rutile phase junction showed poor PEC activity due to charge recombination at the boundaries of the particulates.^19^ This implies that a more rational design for the fabrication of phase junctions in film based energy-conversion devices is needed because of the different charge transfer processes in particulate photocatalysts and film devices. In a film-based photoanode, photogenerated holes will participate in the water oxidation reaction at the electrode–electrolyte interface and the photogenerated electrons will be pumped away to the cathode for the reduction reaction, while both water oxidation and proton reduction reactions occur on the surface of a single particulate in a photocatalytic system.^19^,^20^ Such reaction configuration differences require the consideration of both charge separation and transportation in a phase junction based photoanode for PEC water splitting.

In order to explicitly demonstrate the functional role of a phase junction in charge separation, the two phases have to be constructed in two separate layer regions in a way matching the band structure for ease of charge transfer and separation, either in anatase/rutile (A/R) or rutile/anatase (R/A) bilayer configurations. Several studies have been performed to quantify the role of a phase junction in TiO_2_ films.^11^,^19^,^21^–^23^ So far, the reported bilayer TiO_2_ films are limited to the readily prepared rutile/anatase configuration with anatase as the external layer, such as deposition of anatase on rutile on the substrates of NaCl crystal,^11^ quartz,^21^ silicon wafer^22^ or simply direct deposition of anatase on rutile single crystal.^23^ Nevertheless, their conclusions are still limited without a reverse structure (rutile on anatase). It is not a simple matter; because of the metastability of anatase phase TiO_2_ upon high temperature calcination the anatase/rutile configuration with rutile as the external layer has not been reported, which is challenging but important in illustrating the role of phase junction in film systems.

In this work, a TiO_2_ electrode is taken as a prototypical model to study the role of phase junction in PEC water splitting. For comparison, pure anatase phase TiO_2_ (TiO_2_-A), rutile phase TiO_2_ (TiO_2_-R) and bilayer TiO_2_ films with different phase structures were fabricated by a novel phase transformation treatment method. The effects of the phase configuration and interface structure of the anatase–rutile phase junctions on PEC performance were systematically investigated using transient absorption spectroscopy, electrochemical and photoelectrochemical measurements. It was found that the TiO_2_-AR film with an appropriately introduced phase junction exhibits much better charge separation and transport properties than those of pure anatase or rutile phase TiO_2_ electrodes, unambiguously demonstrating the advantage of an appropriate phase junction based TiO_2_ film in solar energy conversion applications.

## Results and discussion

**Fig. 1:**
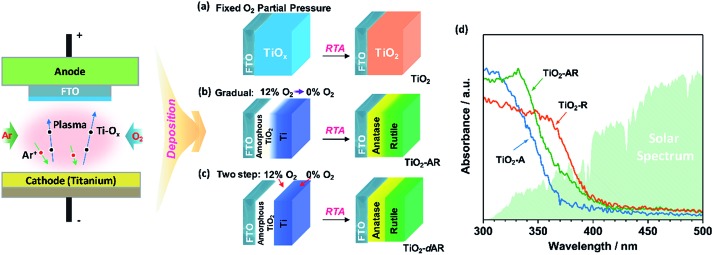
Schematic diagrams illustrating the fabrication of TiO_2_ films with tuneable phase structures using a direct current reactive magnetron sputtering technique followed by rapid thermal annealing (RTA) treatment. (a) Uniform TiO_2_ films including TiO_2_-A and TiO_2_-R were obtained by RTA treatment of the precursor films deposited at a fixed O_2_ partial pressure (≥0%). (b) The TiO_2_-AR film was obtained by RTA treatment of the gradual precursor film deposited by adjusting the O_2_ partial pressure gradually from 12% to 0%. (c) The TiO_2_-dAR film was obtained by RTA treatment of the bilayer precursor film with an internal layer deposited at a fixed 12% O_2_ partial pressure followed by deposition of an external titanium layer at 0% O_2_ partial pressure. (d) The UV-vis absorption spectra of the obtained TiO_2_-A, TiO_2_-R and TiO_2_-AR films.

**Fig. 2:**
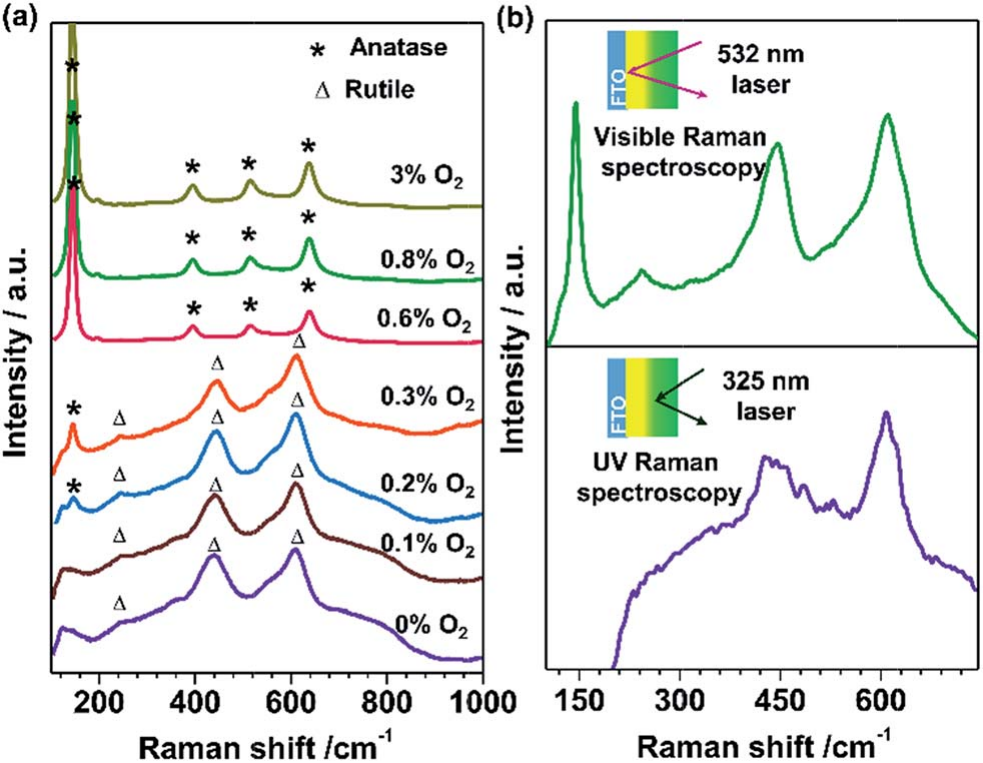
(a) Visible Raman spectra of the TiO_2_ films fabricated at different O_2_ partial pressures. (b) Visible and UV Raman spectra of the TiO_2_-AR electrode, which consists of an internal anatase phase TiO_2_ layer and external rutile phase TiO_2_ layer.

TiO_2_ films with tunable phase structure were fabricated by a novel phase transformation treatment method. The precursor films were firstly deposited on fluorine doped tin oxide (FTO) glass substrates using a direct current reactive magnetron sputtering technique under different fixed oxygen partial pressures (Fig. 1a). The as-deposited films were then subjected to rapid thermal annealing (RTA) treatment at 1073 K for 4 min to obtain crystalline films. The XRD patterns exhibited only diffraction peaks of the FTO glass substrate (Fig. S1a†). Raman spectroscopy was also applied in the study of phase compositions since it is more sensitive to the surface layer of the electrodes than XRD (Fig. S1b†).^24^,^25^ Fig. 2a shows the Raman spectra of TiO_2_ films prepared at different oxygen partial pressures. When the partial pressure of O_2_ was less than 0.1%, Raman peaks at 237, 443 and 610 cm^–1^ attributed to rutile phase TiO_2_ were observed.^25^,^26^ Here, the Raman band at 237 cm^–1^ and the broad background signal at 200–840 cm^–1^ are due to multi-photon scattering.^26^ The obtained rutile film exhibited an optical absorption edge at *ca.* 402 nm (Fig. 1d). Upon elevating the O_2_ partial pressure to 0.2%, a Raman peak at 144 cm^–1^ attributed to anatase phase TiO_2_ appeared simultaneously in addition to the presence of Raman peaks attributed to rutile phase TiO_2_. By further increasing the O_2_ partial pressure up to 0.8%, Raman peaks attributed to rutile phase TiO_2_ completely disappeared and only Raman peaks of anatase phase TiO_2_ at 144, 399, 515 and 519 cm^–1^ were observed. The obtained anatase film exhibited light absorption with an optical absorption edge at 373 nm (Fig. 1d). The results demonstrate that the phase transformation process is largely dependent on the oxygen partial pressure during the sputtering of the precursor films. In other words, precursor films deposited in an oxygen-deficient atmosphere (including the titanium film deposited at 0% O_2_) were readily oxidized into the rutile TiO_2_ phase, while the amorphous TiO_2_ films deposited in an oxygen-rich atmosphere were firstly crystallized into anatase phase in a short calcination time. Note that the phase transformation from anatase to rutile phase is a lengthy process,^6^ which remarkably can be avoided here by using a rapid thermal treatment. Similar results were obtained when precursor films on different substrates (Fig. S2†) were subject to post-calcination treatment at different temperatures (Fig. S3a and b†). This interesting finding allowed us to tune the phase structures of TiO_2_ films.

The TiO_2_ bilayer films consisting of an internal anatase phase TiO_2_ layer and external rutile phase TiO_2_ layer were then fabricated. Firstly, the precursor film was deposited by adjusting the O_2_ partial pressure gradually from 12% to 0% during the deposition process (Fig. 1b). After RTA treatment, the internal amorphous TiO_2_ deposited in an oxygen-rich atmosphere was crystallized into anatase phase TiO_2_, and the external zone deposited in an oxygen-deficient atmosphere was oxidized into rutile phase TiO_2_. The obtained TiO_2_ film was denoted as TiO_2_-AR. The spatial distribution of anatase and rutile phases in TiO_2_-AR film was studied using visible and UV Raman spectra (Fig. 2b), because it is well known that UV Raman spectroscopy is relatively more sensitive to the external region than visible Raman spectroscopy because the absorption of TiO_2_ is only in the UV region (Fig. 1d).^24^,^25^ The optical absorption edge of the TiO_2_-AR film lies in the middle of those of pure anatase and rutile (Fig. 1d), which is due to its composition of anatase and rutile. In contrast to the precursor film deposited by adjusting the O_2_ partial pressure gradually from 12% to 0%, the bilayer precursor film was fabricated by stepwise deposition of an internal amorphous TiO_2_ layer at a fixed 12% O_2_ partial pressure followed by deposition of the external titanium layer at 0% O_2_ partial pressure (Fig. 1c). After RTA treatment, the internal layer was crystallized into anatase phase TiO_2_ and the external titanium layer was oxidized into rutile phase TiO_2_. The obtained bilayer TiO_2_ was denoted as TiO_2_-dAR. Overall, two different TiO_2_ electrodes in an anatase/rutile configuration with rutile as the external layer have been successfully fabricated. The TiO_2_-RA electrode with reverse phase configuration with the anatase phase TiO_2_ as the external layer was also fabricated (see details in the ESI†).

**Fig. 3:**
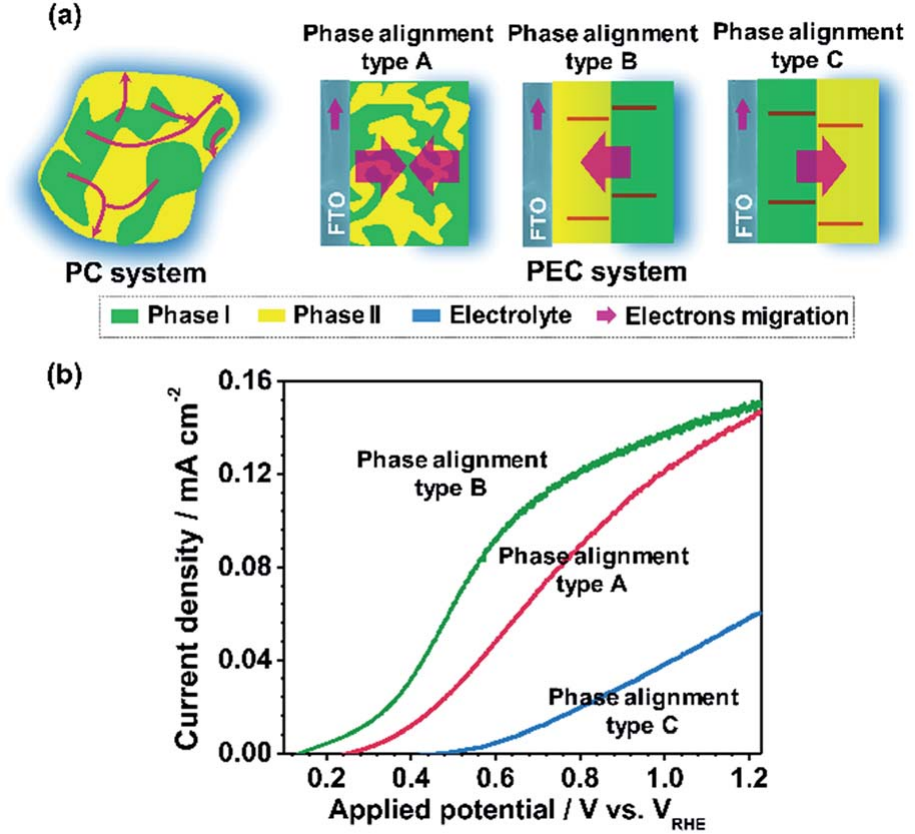
(a) Schematic diagrams showing the phase junction effects on charge separation and transfer in PC system and PEC system. Type A is the electrode with random phase alignment. Type B and type C are the electrodes with phase alignments for forward and reverse electron migration, respectively. (b) *J*–*V* curves of TiO_2_ electrodes with type A (red), type B (green) and type C (blue) phase alignments, representing the TiO_2_ electrodes deposited at 0.3% O_2_, the TiO_2_-dAR electrode and the TiO_2_-RA electrode, respectively.

For the basis of comparison, the thicknesses of TiO_2_-R (deposited at 0% O_2_), TiO_2_-A (deposited at 3% O_2_) and mixed phase TiO_2_ (random phase alignment, deposited at 0.3% O_2_) photoelectrodes were deliberately controlled to be *ca.* 80 nm (Fig. S4a†). The TiO_2_-R electrode exhibited an onset potential (*V*_onset_) of *ca.* 0.30 V *versus* RHE (*V*_RHE_) and a photocurrent density of *ca.* 0.07 mA cm^–2^ at 1.23*V*_RHE_ (Fig. S4b†). However, the TiO_2_-A showed a negatively shifted *V*_onset_ of *ca.* 0.16*V*_RHE_ and a higher photocurrent density of *ca.* 0.21 mA cm^–2^ at 1.23*V*_RHE_. Furthermore, the performance of the mixed phase TiO_2_ falls between those of TiO_2_-A and TiO_2_-R electrodes with a *V*_onset_ of *ca.* 0.28*V*_RHE_ and a photocurrent density of *ca.* 0.15 mA cm^–2^ at 1.23*V*_RHE_. This is not controversial in the promotion of charge separation by the anatase–rutile junction in mixed phase TiO_2_ powder,^6^,^27^ if one considers the different charge transport modes between PC and PEC water splitting processes (Fig. 3a). In a PC system, the photogenerated electrons and holes separated across the phase junction can readily react with the reactants on the surface, while in a PEC system, the photogenerated charges have to be transported in the desired direction.^19^,^20^ Thus, an ordered alignment of the different phases in an appropriate band structure configuration favoring charge transfer between different phases is essential for unidirectional charge transfer in a PEC system. Obviously, both the random alignment of the different phases and the ordered phase alignment of different phases but in a reversed band structure configuration could all increase the possibility of electron–hole recombination, leading to inefficient charge separation and hence low PEC performance.

Fig. 3b shows the *J*–*V* curves of the TiO_2_ electrodes in three different types of phase alignment configurations. It can be seen that the photocurrent densities are in the order of type B > type A > type C at 0.8*V*_RHE_. Herein, taking the TiO_2_ electrodes of type A, type B and type C as a demonstration, the effect of the anatase–rutile phase junction on onset potential can be depicted. Ideally, the occurrence of a photocurrent requires that the sum of the applied potential (*E*_app_), phase junction potential (*E*_j_) and electrode–electrolyte solid–liquid junction potential (*E*_ee_) should be at least larger than the activation energy (*E*_a_) of water oxidation on the surface of the semiconductor under illumination as shown in the following expression:*E*_app_ + *E*_j_ + *E*_ee_ ≥ *E*_a_.

When the water oxidation reaction is just about to start, the above equation can be expressed as *V*_onset_ + *E*_j_ + *E*_ee_ = *E*_a_. At the equilibrium state, the TiO_2_ electrode exhibits upward band bending due to its n-type character. As shown in Fig. 3b, the TiO_2_ electrode in type B phase alignment configuration, where the vector direction of *E*_j_ is same as that of *E*_ee_, exhibits the lowest *V*_onset_ of *ca.* 0.15*V*_RHE_. If the TiO_2_ electrode is in type C phase alignment configuration where the vector direction of *E*_j_ is reverse to that of *E*_ee_, it exhibits the largest onset potential of *ca.* 0.48*V*_RHE_. In the TiO_2_ electrode with type A configuration where the vector direction of *E*_j_ is uncertain due to random phase alignment, it exhibits a moderate onset potential of *ca.* 0.27*V*_RHE_. Therefore, the appropriate phase alignment of a phase junction is vitally important in a PEC system to minimize the onset potential and achieve efficient charge collection.

**Fig. 4:**
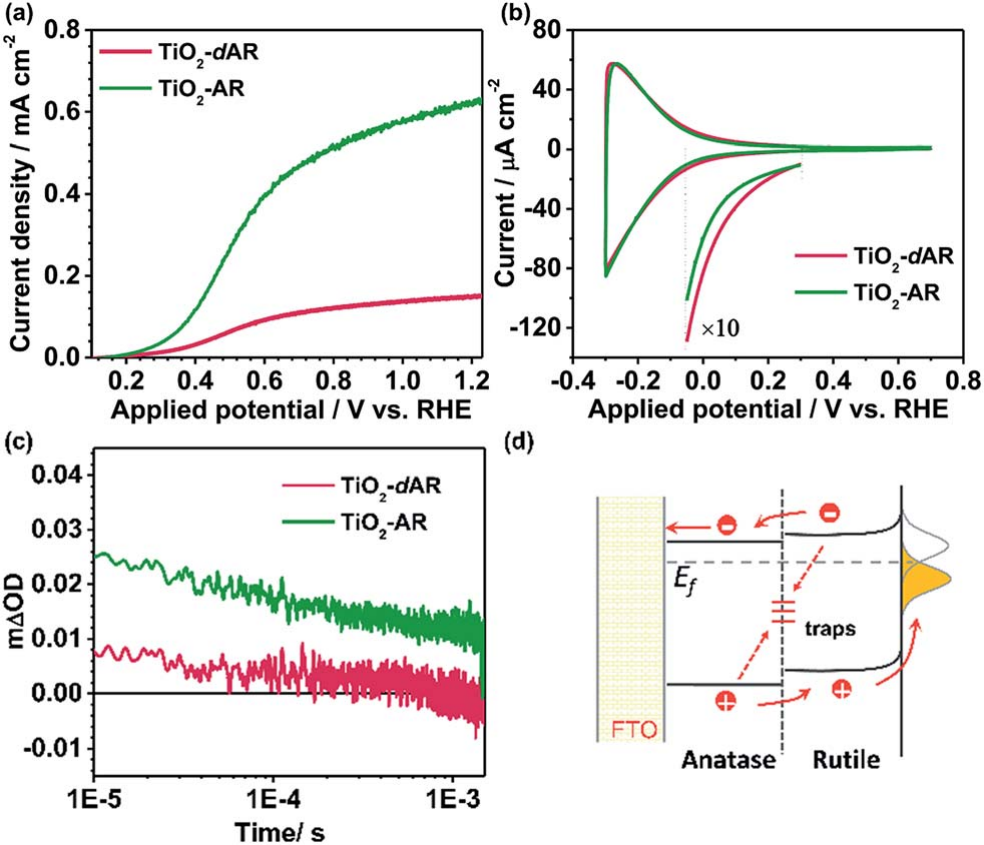
(a) *J*–*V* curves of TiO_2_-dAR (red) and TiO_2_-AR (green). (b) Cyclic voltammograms curves of TiO_2_-dAR (red) and TiO_2_-AR (green) in N_2_-purged 0.1 M HClO_4_. Scan rate: 50 mV s^–1^. (c) Transient absorption decay profiles of TiO_2_-dAR and TiO_2_-AR on the μs–ms timescale probed with a 550 nm excitation line at room temperature. A 75 W tungsten lamp and a Nd:YAG laser (355 nm) were used as the probe and the pump beam, respectively. (d) Schematic diagrams showing the possible charge transfer processes across the phase junction. The dotted arrows represent undesirable interface trapping/recombination processes.

Fig. 4a shows the *J*–*V* curves of TiO_2_-AR and TiO_2_-dAR. It can be seen that the TiO_2_-AR electrode exhibits a photocurrent density of *ca.* 0.63 mA cm^–2^ at 1.23*V*_RHE_, which is 3 and 9 times those obtained for the TiO_2_-A and TiO_2_-R electrodes, respectively. Moreover, the onset potential is negatively shifted to *ca.* 0.15*V*_RHE_. The drastic enhancement of TiO_2_-AR in terms of PEC water splitting performance verifies the importance of ordered phase alignment for achieving efficient charge separation and collection in a PEC system. However, the TiO_2_-dAR electrode exhibits a photocurrent density of only *ca.* 0.15 mA cm^–2^ at 1.23*V*_RHE_ (Fig. 4a), which is much lower than that of the TiO_2_-AR electrode though both of them are in the same anatase/rutile phase alignment (Fig. 1b and c). The origin of such a dramatic performance difference may arise from the difference in their junction structures.^28^–^30^ Xia *et al.* reported that the mismatched electronic structures and the following distortion and defects between the anatase and rutile interface affect charge transport and transfer across the interface.^30^ In the fabrication process of TiO_2_-dAR, the stepwise deposition of amorphous TiO_2_ and titanium could bring about a rigid interface (Fig. 1c), leading to serious interfacial dislocation at junctions. The interfacial dislocation is likely to produce additional states at the phase junction interface to generate both a conduction band energy barrier for electron transfer and recombination centers for excess minority carriers.^28^ In TiO_2_-AR, the phase junction introduced by the gradual deposition method may largely relax such interface dislocations (Fig. 1b). The above argument was confirmed by cyclic voltammetry (CV) experiments as shown in Fig. 4b. The relatively symmetrical shape of the CVs reflects the capacitive nature of the electronic states either at the conduction band or sub-band states located below it. Energetically, electrons tend to fill the sub-band states at about *E* < 0.3*V*_RHE_ first and then the conduction band of TiO_2_ at about *E* < 0.1*V*_RHE_ during the charging process.^31^ It can be seen that both TiO_2_-dAR and TiO_2_-AR show a higher charging current than those of pure phase TiO_2_ films (Fig. S5†) due to filling of the sub-band states introduced by the anatase–rutile interface. The relatively lower charging current of TiO_2_-AR compared to that of TiO_2_-dAR is an indication that the TiO_2_-AR possesses less sub-band states. Such sub-band states may act as charge recombination centers to reduce charge separation efficiency and/or charge carrier mobility, which may account for the differences in the observed PEC performances (Fig. 4a). As a consequence, the conduction band energy barrier in TiO_2_-AR is lower than that in TiO_2_-dAR. The possible charge transfer processes across the phase junction are depicted in Fig. 4d. In addition to the phase alignment, the interfacial electronic structure is also a crucial factor in deliberating the charge separation and transfer potential of the anatase–rutile phase junction.

The dynamics of long-lived photogenerated holes for water oxidation was further studied by transient absorption (TA) spectroscopy. Fig. 4c shows the TA decays profiles of TiO_2_-dAR and TiO_2_-AR with the same film thickness. The transient absorption at 550 nm can be assigned to the long-lived holes.^32^ The yields of long-lived holes under illumination are in the order TiO_2_-AR > TiO_2_-dAR, which is consistent with the results of the PEC performance. The higher yield of long-lived holes in the TiO_2_-AR film is strong evidence that the phase junction introduced by the gradual deposition method facilitates charge separation and transfer.

**Fig. 5:**
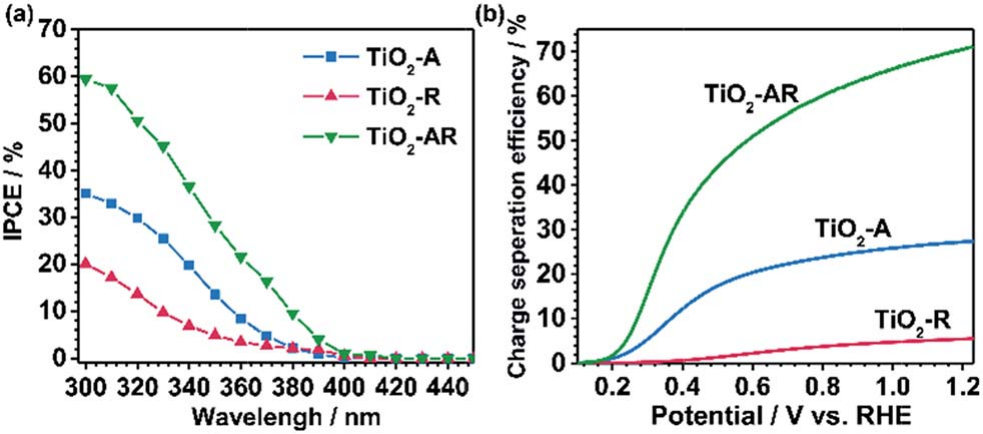
(a) The incident photon-to-electron conversion efficiency (IPCE) values of TiO_2_-A (blue), TiO_2_-R (red) and TiO_2_-AR (green). (b) The carrier separation efficiencies of TiO_2_-A (blue), TiO_2_-R (red) and TiO_2_-AR (green).

The incident photon-to-electron conversion efficiency (IPCE) value of TiO_2_-AR (at 300 nm, 1.23*V*_RHE_) reaches *ca.* 59% (the apparent photon-to-electron conversion efficiency achieves a value of *ca.* 92%) which is *ca.* 2 and 4 times those obtained for TiO_2_-A and TiO_2_-R (Fig. 5a), respectively. This is in good agreement with the results of the photocurrent density measurements. Such a dramatic PEC performance difference cannot be accounted for by morphology differences, since all of the samples show similar morphology and roughness as revealed by the corresponding SEM and AFM images (Fig. S6a–f†). In XPS spectra (Fig. S7†), only the Ti 2p peaks are observed at 485.5 and 464.3 eV which are assigned to Ti^4+^, indicating that no Ti^3+^ species exist on the top layer of the TiO_2_ films. Electron paramagnetic resonance spectra (EPR) recorded at 100 K were also used to test for the presence of Ti^3+^ in our samples. The observation of very weak Ti^3+^ EPR signals with similar intensities also demonstrates that the three electrodes possess similar trace amounts of Ti^3+^ defects probably in the bulk (Fig. S8†). The XPS and EPR results both show that the PEC performance difference cannot be accountable for the valence state difference of titanium. Electrochemical impedance spectroscopy (EIS) (Fig. S9†) shows that the TiO_2_-AR exhibits a much smaller arc radius than TiO_2_-A and TiO_2_-R, indicating that charge transfer kinetics is much faster in TiO_2_-AR electrode (the optimized fitting values are provided in Table S1†).^33^ Both the IPCE and EIS results demonstrate the advantages of TiO_2_-AR for charge separation and transfer in water oxidation reaction.

The PEC performance of the photoanodes was further evaluated by quantitative calculation of the carrier separation efficiencies. The charge separation efficiency (*η*_sep_) is obtained by *η*_sep_ = (*J*_abs_ × *η*_ox_)/*J*_PEC_, where *J*_abs_ is the theoretical maximum photocurrent, *J*_PEC_ is the measured photocurrent density, and *η*_ox_ is the yield of the surface reaching holes that are injected into the solution to participate in the oxidation reaction.^34^,^35^ Sodium sulfite (Na_2_SO_3_) was used to titrate the charge separation efficiency, because the surface recombination of charges can be regarded to be nearly completely suppressed in the presence of Na_2_SO_3_ and thus the *η*_ox_ could be assumed to be 100%. *J*_abs_ values of the photoanodes were calculated using a trapezoidal integration of the absorption spectra by assuming 100% absorbed photon-to-current conversion efficiency. The TiO_2_-AR achieves *η*_sep_ = 0.16 at 0.3*V*_RHE_, which is *ca.* 3 and 60 times those obtained for TiO_2_-A and TiO_2_-R, respectively, as shown in Fig. 5b. The significant enhancement in charge separation achieved at a small bias for TiO_2_-AR is ascribed to the well-defined anatase–rutile phase junction.

**Fig. 6:**
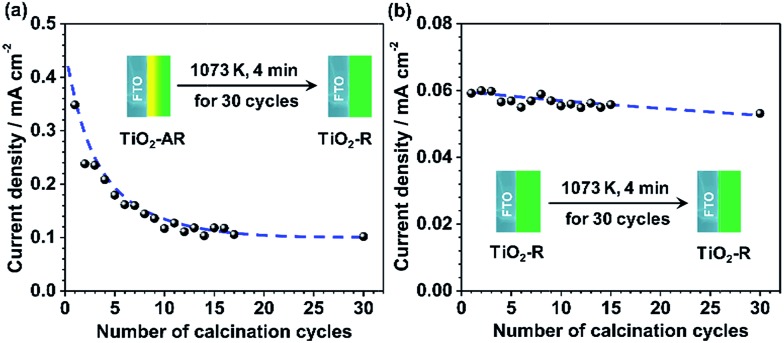
Dependence of the current density values of TiO_2_-AR electrode (a) and TiO_2_-R electrode (b) on the number of calcination cycles. After calcination treatment at 1073 K for 120 min (4 minutes per cycle and 30 cycles), the TiO_2_-AR was converted to pure rutile phase.

In order to further demonstrate the effect of phase junction on charge separation and transfer, the PEC performance of the junction-removed TiO_2_-AR electrode was also examined. The phase junction in TiO_2_-AR was removed deliberately by gradual phase transformation of the internal anatase to rutile *via* 30 cycles of the calcination treatments at 1073 K (4 minutes per cycle). In the end, the TiO_2_-AR was completely transformed into pure rutile phase TiO_2_. The changes in the photocurrent values of TiO_2_-AR at 0.65*V*_RHE_*versus* the calcination cycles are shown in Fig. 6a. It can be seen that the photocurrent decreases with an increase in the number of calcination cycles, from *ca.* 0.35 mA cm^–2^ to *ca.* 0.1 mA cm^–2^. This indicates that the simultaneous existence of both anatase and rutile is a prerequisite for efficient charge separation and transfer. In other words, it is the anatase–rutile phase junction that leads to the higher charge separation efficiency therefore the better PEC performance of the TiO_2_-AR electrode in water splitting. The possible cause of photocurrent decrease being FTO destruction can be ruled out, since a negligible photocurrent decrease was observed for the TiO_2_-R electrode which was subjected to the same treatment (Fig. 6b). These results strongly support our claim that the improvement of the PEC performance of TiO_2_-AR photoanode is unambiguously due to the well-defined anatase–rutile phase junction.

## Conclusions

In summary, a novel phase transformation method by rapid thermal annealing treatment has been employed to tune the anatase and rutile phase configurations and compositions in TiO_2_ thin film photoanodes. A synthetically challenging TiO_2_ photoanode with an anatase/rutile configuration has been fabricated, which allowed us to study the phase junction configuration effect on charge separation and transfer in thin film-based devices for the first time. It was found that the performance of the phase junction based devices is extremely sensitive to the phase configuration and interface structure. A random phase alignment in mixed phase TiO_2_ increases the possibility of electron–hole pair recombination, leading to inefficient charge separation and low PEC water splitting activity. An ordered phase alignment with an abrupt phase junction in TiO_2_ film could increase the charge separation and transfer efficiency by only a limited level due to the existence of relatively serious interfacial dislocation and large conduction band energy barrier. However, the TiO_2_ film with an appropriate phase junction introduced by a gradual deposition method showed superior performance in charge separation and transfer, hence achieving 3 and 9 times photocurrent density enhancement at 1.23*V*_RHE_ compared to the pristine anatase and rutile phase TiO_2_ electrodes, respectively. The onset potential of TiO_2_-AR was only 0.15*V*_RHE_, which was negatively shifted by 150 mV compared to that of the pristine rutile phase TiO_2_. The key to phase junction engineering in TiO_2_ films for efficient charge separation and transfer is to simultaneously consider both the phase alignment for the unidirectional charge transfer and the interface structure to minimize the interface trap states. This work not only provides more in-depth understanding of the phase junction effect in TiO_2_ film-based devices, but also reveals that a TiO_2_ film with a precisely tuned phase junction may hold great promise in energy conversion applications.

## Supplementary Material

Supplementary information

## References

[cit1] Chen X., Li C., Gratzel M., Kostecki R., Mao S. S. (2012). Chem. Soc. Rev..

[cit2] Ma Y., Wang X., Jia Y., Chen X., Han H., Li C. (2014). Chem. Rev..

[cit3] Jang J. S., Kim H. G., Lee J. S. (2012). Catal. Today.

[cit4] Hong S. J., Lee S., Jang J. S., Lee J. S. (2011). Energy Environ. Sci..

[cit5] Chen S., Qi Y., Hisatomi T., Ding Q., Asai T., Li Z., Ma S. S., Zhang F., Domen K., Li C. (2015). Angew. Chem., Int. Ed..

[cit6] Zhang J., Xu Q., Feng Z., Li M., Li C. (2008). Angew. Chem., Int. Ed..

[cit7] Wang X., Xu Q., Li M., Shen S., Wang X., Wang Y., Feng Z., Shi J., Han H., Li C. (2012). Angew. Chem., Int. Ed..

[cit8] Fujishima A., Honda K. (1972). Nature.

[cit9] Hurum D. C., Agrios A. G., Gray K. A., Rajh T., Thurnauer M. C. (2003). J. Phys. Chem. B.

[cit10] Ohno T., Tokieda K., Higashida S., Matsumura M. (2003). Appl. Catal., A.

[cit11] Scanlon D. O., Dunnill C. W., Buckeridge J., Shevlin S. A., Logsdail A. J., Woodley S. M., Catlow C. R., Powell M. J., Palgrave R. G., Parkin I. P., Watson G. W., Keal T. W., Sherwood P., Walsh A., Sokol A. A. (2013). Nat. Mater..

[cit12] Kavan L., Grätzel M., Gilbert S. E., Klemenz C., Scheel H. J. (1996). J. Am. Chem. Soc..

[cit13] Ong S. W. D., Lin J., Seebauer E. G. (2015). J. Phys. Chem. C.

[cit14] Park N. G., van de Lagemaat J., Frank A. J. (2000). J. Phys. Chem. B.

[cit15] Todinova A., Idigoras J., Salado M., Kazim S., Anta J. A. (2015). J. Phys. Chem. Lett..

[cit16] Lee S., Noh J. H., Han H. S., Yim D. K., Kim D. H., Lee J. K., Kim J. Y., Jung H. S., Hong K. S. (2009). J. Phys. Chem. C.

[cit17] Choi B. J., Jeong D. S., Kim S. K., Rohde C., Choi S., Oh J. H., Kim H. J., Hwang C. S., Szot K., Waser R., Reichenberg B., Tiedke S. (2005). J. Appl. Phys..

[cit18] Tian Y., Tatsuma T. (2005). J. Am. Chem. Soc..

[cit19] Wang X., Jin S., An H., Wang X., Feng Z., Li C. (2015). J. Phys. Chem. C.

[cit20] Wang Z. Qi Y. Ding C. Fan D. Liu G. Zhao Y. Li C., Chem. Sci., 201610.1039/c6sc00245e , , accepted manuscript . 10.1039/c6sc00245e PMC6014074 30155086

[cit21] Kawahara T., Konishi Y., Tada H., Tohge N., Nishii J., Ito S. (2002). Angew. Chem., Int. Ed..

[cit22] Chung C. K., Liao M. W., Lai C. W. (2009). Thin Solid Films.

[cit23] Pfeifer V., Erhart P., Li S., Rachut K., Morasch J., Brötz J., Reckers P., Mayer T., Rühle S., Zaban A., Mora Seró I., Bisquert J., Jaegermann W., Klein A. J. (2013). J. Phys. Chem. Lett..

[cit24] Li M., Feng Z., Ying P., Xin Q., Li C. (2003). Phys. Chem. Chem. Phys..

[cit25] Zhang J., Li M., Feng Z., Chen J., Li C. (2006). J. Phys. Chem. B.

[cit26] Frank O., Zukalova M., Laskova B., Kurti J., Koltai J., Kavan L. (2012). Phys. Chem. Chem. Phys..

[cit27] Ma Y., Wang X., Li C. (2015). Chin. J. Catal..

[cit28] Oldham W. G., Milnes A. G. (1964). Solid-State Electron..

[cit29] Zhao W., Zhu S., Li Y., Liu Z. (2015). Chem. Sci..

[cit30] Xia T., Li N., Zhang Y., Kruger M. B., Murowchick J., Selloni A., Chen X. (2013). ACS Appl. Mater. Interfaces.

[cit31] Berger T., Lana-Villarreal T. (2007). J. Phys. Chem. C.

[cit32] Wang X., Kafizas A., Li X., Moniz S. J. A., Reardon P. J. T., Tang J., Parkin I. P., Durrant J. R. (2015). J. Phys. Chem. C.

[cit33] Liu H., Li X., Leng Y., Li W. (2003). J. Phys. Chem. C.

[cit34] Dotan H., Sivula K., Grätzel M., Rothschild A., Warren S. C. (2011). Energy Environ. Sci..

[cit35] Yan P., Liu G., Ding C., Han H., Shi J., Gan Y., Li C. (2015). ACS Appl. Mater. Interfaces.

