# Supplementary material for: Understanding the anatase–rutile phase junction in charge separation and transfer in a TiO2 electrode for photoelectrochemical water splitting
Source: Chem Sci. 2016 Jun 9;7(9):6076–82. doi: 10.1039/c6sc01611a (PMC6022233; doi:10.1039/c6sc01611a)
Supplement: Supplementary file 1 [file SC-007-C6SC01611A-s001.pdf]

Electronic Supplementary Material (ESI) for Chemical Science.
This journal is © The Royal Society of Chemistry 2016

Supporting Information

Anatase-Rutile Phase Junction on Charge Separation and Transfer in TiO_2 Electrode for Photoelectrochemical Water Splitting

Ailong Li,[ab] Zhiliang Wang,[ab] Heng Yin,[ab] Shengyang Wang,[ab] Pengli Yan,[ab] Baokun Huang,[a] Xiuli Wang,[a] Rengui Li,[a] Xu Zong,[a] Hongxian Han,[ac*] Can Li[ac*]

[a] State Key Laboratory of Catalysis, Dalian Institute of Chemical Physics, Chinese Academy of Sciences; Dalian National Laboratory for Clean Energy, Dalian, 116023, China.

[b] Graduate University of Chinese Academy of Sciences, Beijing 100049, China

[c] Collaborative Innovation Center of Chemistry for Energy Materials (*i*ChEM)

[*]Email of corresponding author: hxhan@dicp.ac.cn, canli@dicp.ac.cn

Experimental Section

Material synthesis

TiO_2 films with tunable phase structure were fabricated using direct-current reactive magnetron sputtering technique. This method includes two main steps: deposition of precursor film and rapid thermal annealing (RTA) treatment. TiO_2 precursor films were first deposited on commercial FTO glass (Nippon Sheet Glass, Japan, sheet resistance ca. 14 ohm per square, FTO coating thickness ca. 350nm, glass thickness ca. 2.2 mm) by the DC reactive physical vapor deposition (PVD) method using a commercial sputtering system equipped with a turbo molecular pump. A titanium metal disk (99.995% purity, 3 mm thickness, 60 mm diameter) was used as the target. After evacuation to 10^{-3} Pa, the argon and oxygen gases were introduced into the chamber respectively through the mass flow controller. The oxygen outlet is over the target about 40mm. The total sputtering pressure was kept at 2.0 Pa with the oxygen partial pressure in the range of 0-15% ($PO_2\% = PO_2 / (PO_2+PAr) \times 100\%$). A constant current mode was used and the sputtering current was kept at 0.6 A. The distance between the target and the sample holder was 60 mm. The titanium deposition rate was about 40 nm/min. At 12% O_2 partial pressure, the deposition rate of amorphous TiO_2 was about 8 nm/min. A corundum boat was used as the sample holder during the annealing procedure. The resulting films were annealed under air atmosphere in a muffle furnace preheated to a certain temperature.

The TiO_2-AR film with gradually introduced phase junction was fabricated by adjusting the O_2 partial pressures gradually from 12% to 0% during the deposition process. The deposition time for the TiO_2-AR electrode with 80 nm thickness was ca.130 s. After rapid

thermal annealing (RTA) treatment, the internal amorphous TiO_2 zone of the film deposited at oxygen-rich conditions was crystallized into anatase phase TiO_2, and the external zone deposited at oxygen-deficient conditions was oxidized into rutile phase TiO_2.

The TiO_2-*d*AR with abrupt phase junction was fabricated by deposition of the internal amorphous TiO_2 layer at oxygen-rich conditions (12% O_2) first, followed by deposition of the external titanium layer at oxygen-free conditions (0% O_2). After RTA treatment, the internal layer was crystallized into anatase phase TiO_2 and the external titanium layer was oxidized into rutile phase TiO_2.

For comparison, the TiO_2-RA electrode was also fabricated. The titanium layer was firstly deposited on the FTO substrate, calcined at 1073K for 4min, followed by the amorphous TiO_2 layer deposition at 12% O_2 and calcinations treatment at 1073 k for 4 min.

Characterization

Raman: The anatase and rutile phase was verified by visible Raman and UV Raman spectroscopy recorded on a Renishaw inVia Raman microscope. Single-frequency laser (532 nm, DPSS 532 Model 200) was used as the exciting source for the visible Raman with an output of 2 - 10 mW and a He-Cd laser (325 nm) was used as the exciting source for the UV Raman. Need to mention that, if the UV resonance Raman spectra were recorded with the Renishaw inVia Raman microscope with the Rayleigh scattering filter, the Rayleigh scattering lower than 200 cm^{-1} could be filtered from the spectrum. In order to verify this point, we also recorded on UV Raman spectrum with a Jobin-Yvon T64000 triple-stage spectrograph which can detect Raman peaks down to 100 cm^{-1}. The spectrum of FTO-AR shows only three characteristic bands of rutile phase TiO_2 at 247, 443 and 610 cm^{-1} as shown

followed. This confirms the absence of UV Raman scattering features due to either anatase or rutile phase TiO_2 at wavenumber lower than 200 cm^{-1} for our TiO_2-AR electrode. In the article we use the visible Raman spectra and UV Raman spectra recorded on the Renishaw inVia Raman microscope for easy comparison.

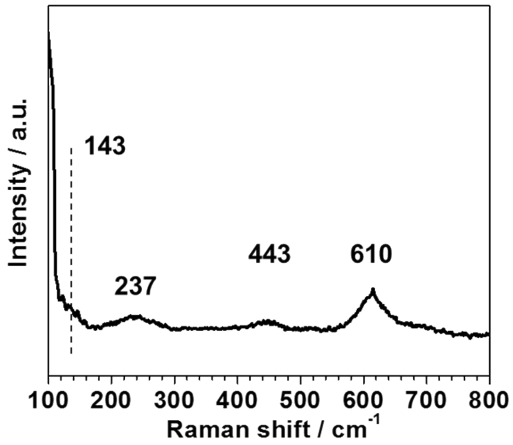

UV Raman spectrum (325 nm) of FTO-AR spectrum recorded with a Jobin-Yvon T64000 triple-stage spectrograph.

The morphology of TiO_2 films were characterized using a field emission scanning electron microscopy (FE-SEM, Quanta 200 FEG). The thicknesses of the obtained films were first measured using a stylus profiler (Dektak XT) and further confirmed by the cross-section SEM images. Atomic force microscopy (AFM) images were measured on a Veeco Nanoscope III-D AFM.

Current-voltage (J-V) curves of the different TiO_2 photoanodes were measured in a three-electrode mode on an electrochemical workstation (CHI760A, Shanghai Chenhua Instruments, Shanghai, China). A Pt plate (20 * 20 * 1 mm) was used as the counter electrode, a saturated calomel electrode (SCE) was employed as the reference electrode, and the fabricated TiO_2 film on FTO was used as the working electrode. The electrolyte was 1 M NaOH (PH = 13.6). Xe lamp (Perfectlight PLX SXE300C, 300 mW cm^{-2}) was used as light

source. Front side illumination was applied for the all the PEC measurements.

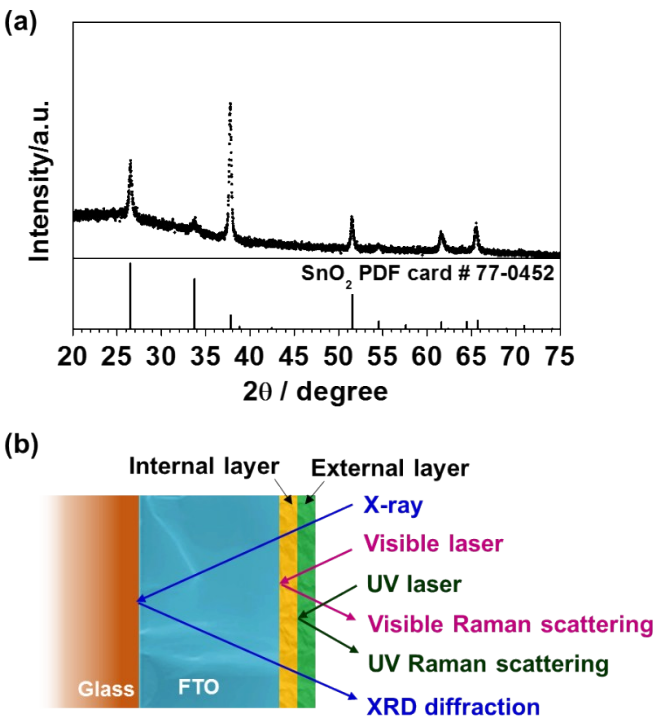

Figure S1. (a) XRD pattern of TiO_2 electrode (TiO_2 thickness ca. 80 nm, FTO coating thickness ca. 350 nm, glass thickness ca. 2.2 mm). The XRD patterns exhibited only the diffraction peaks of FTO glass substrate. That the Raman spectroscopic (both UV and visible) is more sensitive to the surface layer of the electrodes than XRD. (b) Schematic diagram showing different depth-sensitivities of XRD, visible Raman spectroscopy, and UV Raman spectroscopy techniques.

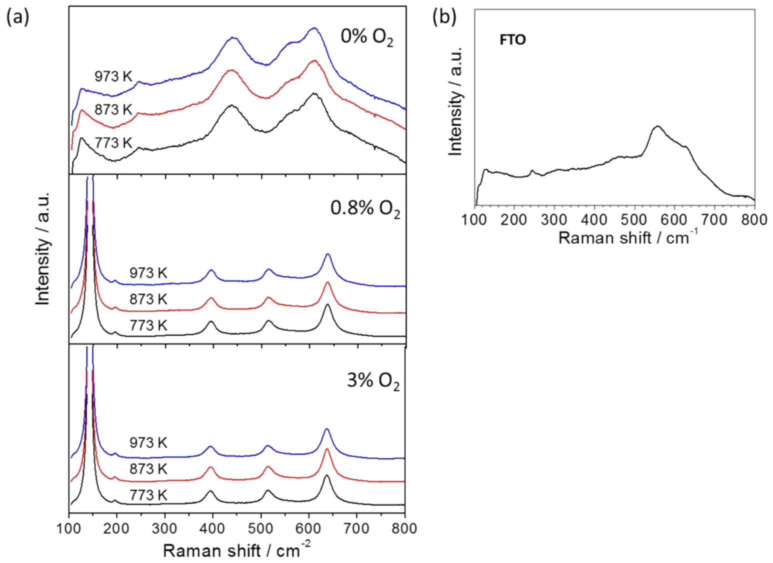

Figure S2. (a) Visible Raman spectra (excitation line at 532 nm) of TiO_2 films fabricated on FTO substrates. (b) Raman spectrum (excitation line at 532 nm) of FTO substrate. Titanium films deposited on FTO substrates at 0% O_2 were oxidized into rutile phase TiO_2 after calcination at 973 K for 5 min, 873 K for 15 min and 773 K for 40 min. Precursor films deposited at 0.8% O_2 and 3% O_2 were transformed into anatase phase TiO_2 after calcination at 973 K for 5 min, 873 K for 15 min and 773 K for 40 min. Precursor films deposited at oxygen-deficient atmosphere (including titanium film deposited at 0% O_2) were oxidized into rutile phase. And amorphous TiO_2 films deposited at oxygen-rich atmosphere were crystallized into anatase phase at relatively short calcination time.

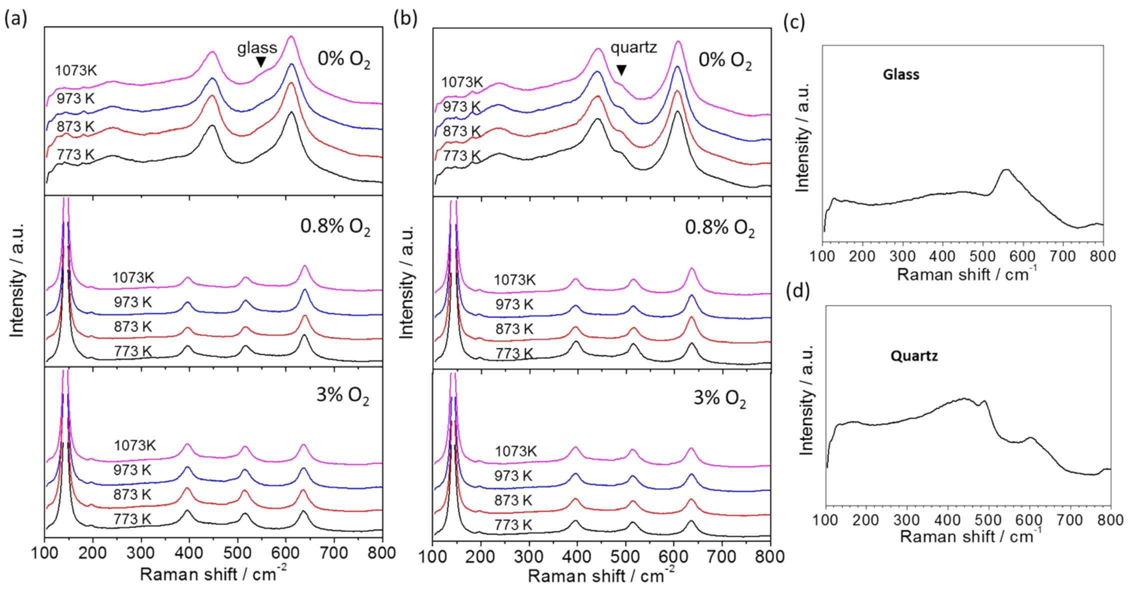

Figure S3. Visible Raman spectra (excitation line at 532 nm) of TiO_2 films fabricated on 2 mm glass sheet (a) and 1 mm quartz (b). The Raman spectra (excitation line at 532 nm) of the glass (c) and quartz substrates (d). Titanium films deposited on 2 mm glass sheet (or 1 mm quartz) substrates at 0% O_2 partial pressure were oxidized into rutile phase TiO_2 after calcination at 973 K for 5 min, 873 K for 15 min and 773 K for 40 min, while the precursor films deposited at 0.8% O_2 and 3% O_2 partial pressures were transformed into anatase phase TiO_2. Precursor films deposited at oxygen-deficient atmosphere (including titanium film deposited at 0% O_2 partial pressrue) were oxidized into rutile phase. And amorphous TiO_2 films deposited at oxygen-rich atmosphere were crystallized into anatase phase at relatively short calcination time.

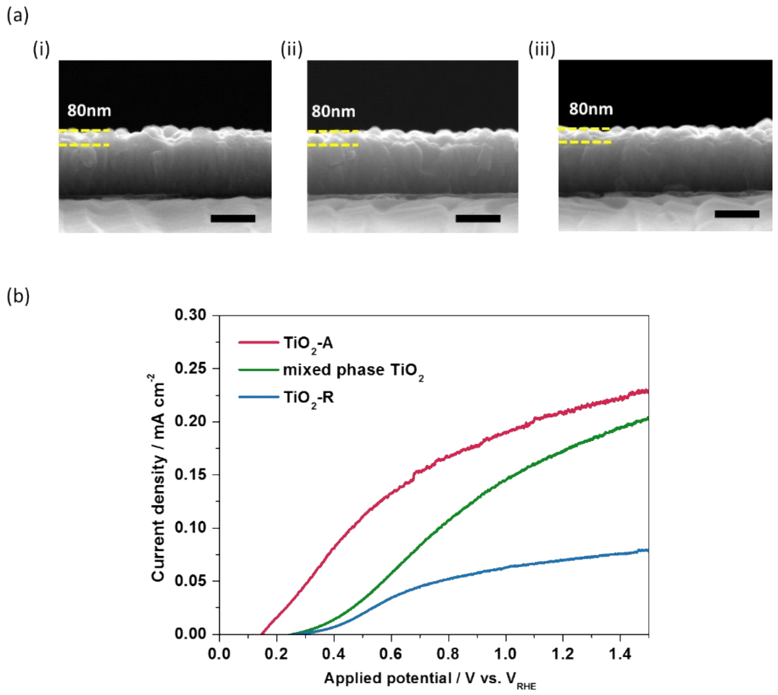

Figure S4. (a) Cross-section scanning electron microscopy (SEM) images of TiO_2-A (i), TiO_2-R (ii) and mixed phase TiO_2 (iii) on FTO substrates. The scale bar is 300nm. (b) J-V curves of TiO_2-A (deposited at 3% O_2 partial pressure), mixed phase TiO_2 (deposited at 0.3% O_2 partial pressure) and TiO_2-R (deposited at 0% O_2 partial pressure). Measurements were performed in 1 M NaOH aqueous solution (pH = 13.6) under Xe lamp illumination (300 mW cm^{-2}).

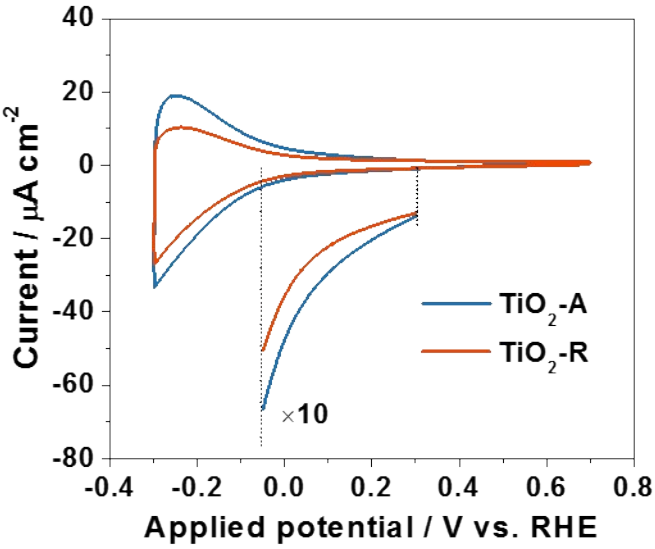

Figure S5. Cyclic voltammograms of TiO_2-A and TiO_2-R in N_2-purged 0.1 M $HClO_4$. Scan rate: 50 mV s^{-1}.

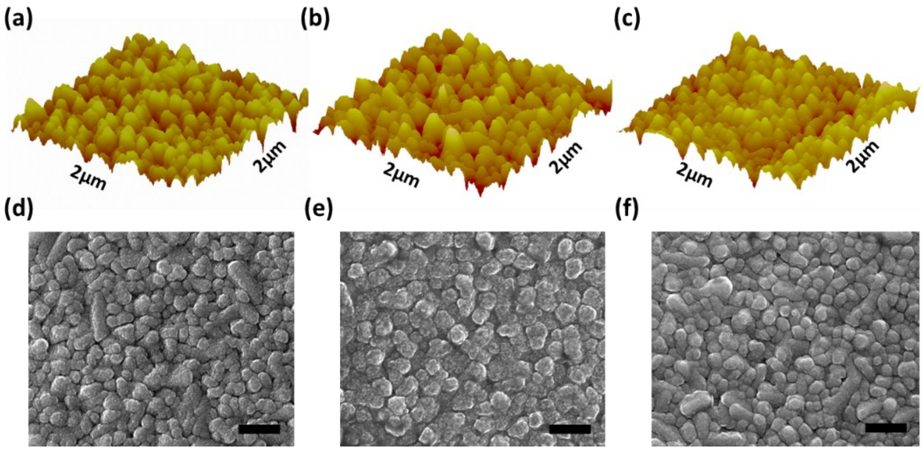

Figure S6. The atomic force microscopy (AFM) images of TiO_2-A (a), TiO_2-R (b) and TiO_2-AR (c) electrodes. The scanning electron microscopy (SEM) images of TiO_2-A (d), TiO_2-R (e) and TiO_2-AR (f) electrodes. The scale bar is 300nm.

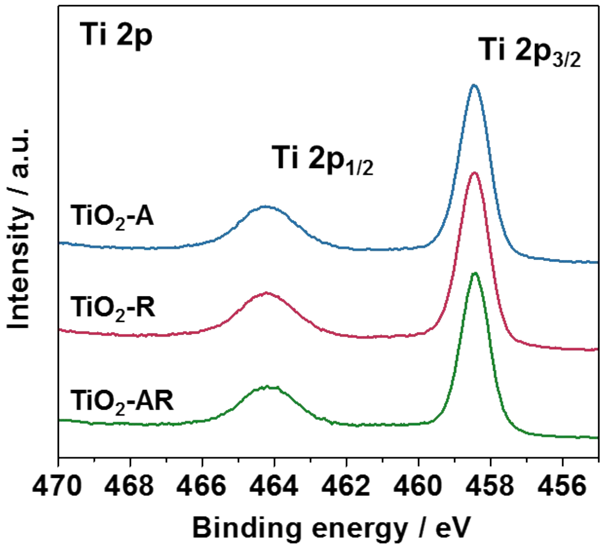

Figure S7. XPS peaks for Ti 2p of different TiO_2 electrodes.

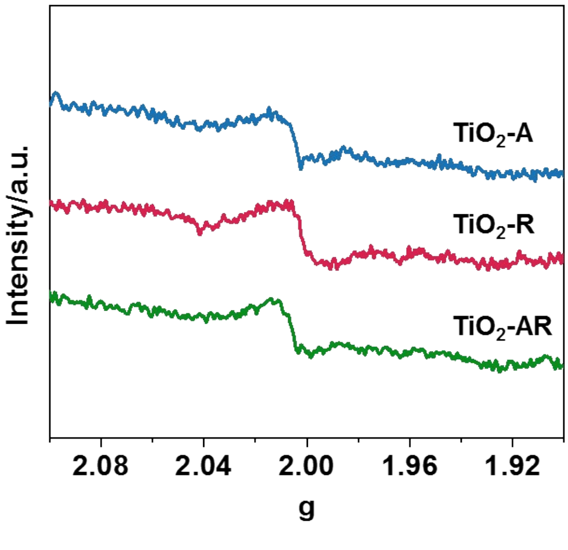

Figure S8. EPR spectra (100 K) of different TiO_2 electrodes.

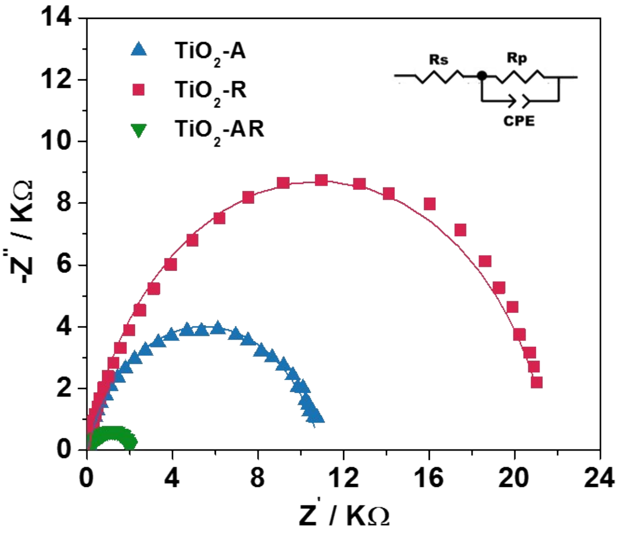

Figure S9. Electrochemical impedance spectra (EIS) recorded as Nyquist plots of TiO_2-A, TiO_2-R and TiO_2-AR electrodes, measured in 1 M NaOH (PH=13.6) at 0.5 V_{RHE}. The experiment data are represented by the discrete points and the simulated responses are shown by solid lines. The inset is the equivalent circuit applied for the simulation.

Table S1. The derived R and CPE values by fitting the Nyquist plots in Figure S8.

	R_s / Ω	Rp / KΩ	CPE-T / μF
A-TiO_2	25	11	7.8
R-TiO_2	59	22	7.9
AR-TiO_2	21	2	28.9
